# Endogenous Expression of the Class I Phosphatidylinositol Transfer Protein PPIT-2 in *Caenorhabditis elegans*

**DOI:** 10.17912/micropub.biology.002386

**Published:** 2026-09-24

**Authors:** Graycen Horne, Xiaofei Bai

**Affiliations:** 1 Department of Biology, University of Florida, Gainesville, FL, United States; 2 Genetics Institute, University of Florida, Gainesville, Florida, United States

## Abstract

Phosphatidylinositol transfer proteins (PITPs) transfer phosphatidylinositol (PI) between the ER and target membranes to regulate phosphoinositide metabolism. However, the expression patterns of class I PITPs in *C. elegans* remain largely uncharacterized. Here, we generated an endogenous GFP knock-in reporter for PPIT-2 (formerly Y71G12B.17), a conserved homolog of human PITPNA and PITPNB. GFP::PPIT-2 was prominently expressed in the excretory system and canals, with additional expression in the hypodermis, sperm, developing vulva, and germline-adjacent pseudocoelom. PPIT-2 expression notably overlaps with fatty acid synthase FASN-1, establishing candidate tissues to investigate its roles in PI transfer, membrane trafficking, and lipid homeostasis.

**Figure 1. Endogenous expression of GFP::PPIT-2 in multiple C. elegans tissues:** (A) Schematic representation of the endogenous *GFP::PPIT-2* reporter locus with an N-terminal GFP tag immediately upstream of the *PPIT-2* coding sequence. (B–B″) Representative images showing GFP::PPIT-2 (green in B'') in the anterior excretory region near the pharynx, co-localization with FASN-1 ::RFP (magenta in B''). Monochromatic images show GFP::PPIT-2 (B) and FASN-1 ::RFP (B'). Yellow arrows highlight representative areas of the spatial overlap. (C–C″) Co-expression of GFP::PPIT-2 (C) and FASN-1 ::RFP (C') in hypodermal cells. with the merged image shown in (C''). Yellow arrows indicate hypodermal nuclei. (D) GFP::PPIT-2 signal in the pseudocoelomic region (yellow arrow) and residual-body-associated structures (red arrow). (E) GFP::PPIT-2 expression in sperm; the yellow arrow points to a representative GFP-positive sperm cell. (F) GFP::PPIT-2 expression in the developing vulval (yellow arrow). Scale bars are indicated in each panel.

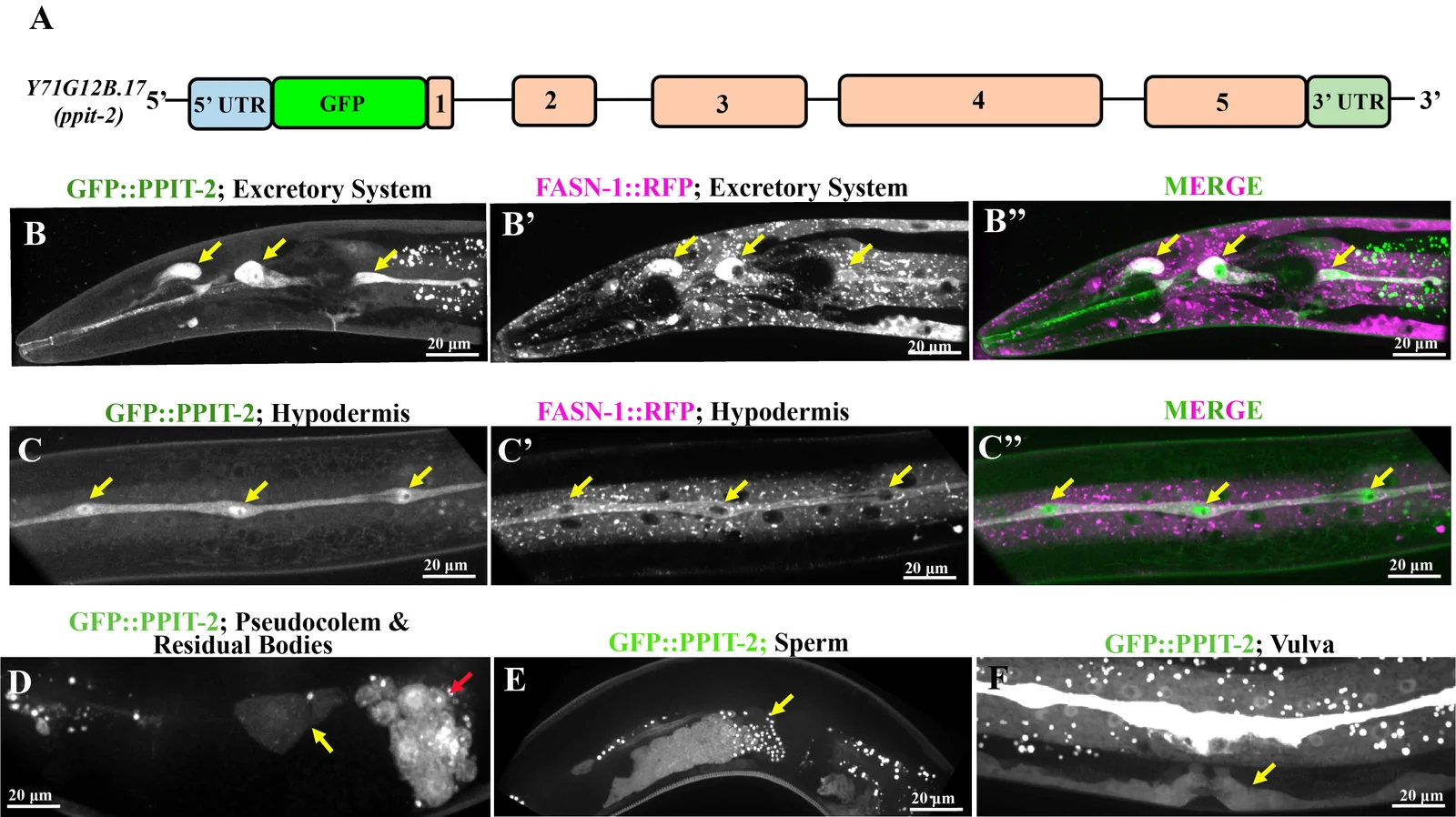



## Description

Phosphatidylinositol transfer proteins (PITPs) are conserved lipid-binding proteins that support phosphatidylinositol (PI) metabolism and phosphoinositide-dependent cellular processes. PI is synthesized primarily in the endoplasmic reticulum (ER), whereas its phosphorylated derivatives are enriched at distinct cellular membranes, such as PI4P at the Golgi complex and PI(4,5)P2 at the plasma membrane (Ashlin, Blunsom, & Cockcroft, 2021). Class I PITPs, including mammalian PITPNA and PITPNB, specifically bind PI and phosphatidylcholine (PC) to facilitate PI phosphoinositide synthesis across distinct membrane compartments (Ashlin, Blunsom, & Cockcroft, 2021; Grabon, Khan, & Bankaitis, 2015). Consequently, PITPs play essential roles in membrane trafficking, secretion, and intracellular signaling (Ashlin, Blunsom, & Cockcroft, 2021). In mammals, PITPNA performs critical physiological functions in secretory tissues. For example, loss of PITPNA in pancreatic beta cells disrupts PI4P production, leading to hyperglycemia and reduced glucose-stimulated insulin secretion, highlighting its potential as a therapeutic target for type 2 diabetes (Yeh et al., 2023).

The *C. elegans* genome encodes two predicted class I PITPs, Y54F10AR.1 and PPIT-2, as well as the class II PITP PITP-1 (Iwata et al., 2011). PITP-1 has been characterized in sensory neurons, where it regulates phosphoinositide-dependent neurotransmission and behavioral plasticity (Iwata et al., 2011). More recently, reduction of *pitp-1* gene expression was also shown to extend lifespan through insulin/IGF-1 and TOR signaling pathways (Lin et al., 2026). In contrast, relatively little is known about the anatomical distribution or physiological functions of class I PITPs in *C. elegans* .

To determine the endogenous expression pattern of PITPs, we generated an N-terminal GFP knock-in reporter at the endogenous locus of PPIT-2 using CRISPR/Cas9 genome editing (Figure A). GFP::PPIT-2 was detected in the excretory cell body and canals, the hypodermis, mature sperm, sperm-associated residual bodies, and the developing vulva, all of which are known for active lipid metabolism. Prominent expression was observed in the anterior excretory system (Figure B-B'), a structure functionally analogous to the mammalian renal system that serves as a hub for fluid balance and metabolic waste elimination. This system relies heavily on phospholipids, such as PI, and their metabolic derivatives to maintain its unique architecture and support intracellular transport pathways (Sundaram & Buechner, 2016).

Given the roles of class I PITPs in lipid transport and phosphoinositide signaling, we asked whether PPIT-2 is expressed in tissues that also express the lipogenic enzyme FASN-1 . We therefore examined GFP::PPIT-2 in animals that also express FASN-1 ::RFP, an endogenous reporter of the *C. elegans* fatty acid synthase required for *de novo* fatty acid synthesis (Starich, Bai, & Greenstein, 2020; Wang et al., 2026). Endogenous GFP-tagged FASN-1 is broadly expressed in somatic tissues, with prominent expression in the hypodermis, excretory duct, and developing vulva, and serves as a key marker for lipid-enriched cellular compartments (Starich, Bai, & Greenstein, 2020). GFP::PPIT-2 showed marked co-localization with FASN-1 ::RFP within several regions surrounding the pharynx and the excretory cell body (Figure B-B''). Strong GFP::PPIT-2 signal was also observed along the lateral body wall in areas corresponding to the hypodermis (Figure C-C''), where it co-localized with FASN-1 ::RFP. These observations, GFP::PPIT-2 and FASN-1 ::RFP signals, were both detected in the examined excretory and hypodermal regions, indicating tissue-level overlap in these regions.

Beyond the excretory system, GFP::PPIT-2 was detected in several reproductive and reproduction-associated tissues (Figure D-F). Fluorescent signals were present in the pseudocoelomic region adjacent to the germline and within structures associated with the residual body (Figure D) as well as in mature sperm (Figure E). Because the pseudocoelomic cavity functions as a key site for lipid transport and exchange between somatic sheath cells and the germline (Perez & Lehner, 2019), this localization suggests potential roles for PPIT-2 in reproductive lipid transport.

Finally, GFP::PPIT-2 fluorescent signal was observed in the developing vulva (Figure F), another tissue where FASN-1 is known to function (Starich, Bai, & Greenstein, 2020; Wang et al., 2026). Vulval morphogenesis requires coordinated cell-cell signaling, extensive membrane remodeling, and active membrane trafficking (Sternberg, 2005). The presence of PPIT-2 in this tissue suggests that this class I PITP might participate in membrane dynamics during organogenesis and *C. elegans* vulvar morphogenesis.

In summary, our endogenous tag reveals the tissue-specific distribution of the previously uncharacterized class I PITP PPIT-2 in *C. elegans .* Its expression in the excretory system, hypodermis, reproductive structures, and developing vulva—combined with its spatial overlap with FASN-1 —provides an anatomical framework for future investigations into its functional roles in phosphoinositide metabolism, membrane trafficking, and organismal lipid homeostasis.

## Methods

**Microscopy**

All fluorescence imaging was performed using a spinning-disk confocal system equipped with a Nikon 60× 1.2 NA water-immersion objective, a Hamamatsu C15440 ORCA-Fusion BT Digital camera, and a Yokogawa CSU-X1 confocal scanner unit. Imaging acquisition was controlled by Nikon's NIS-Element software (Version 6.10). Image processing and channel merging were conducted using the ImageJ/FIJI Bio-Formats plugin (Fiji/ImageJ: ImageJ version 1.54f; Bio-Formats plugin: version 7.0.0) (National Institutes of Health) (Linkert et al., 2010; Schindelin et al., 2012).

**Mounting *C. elegans* for imaging**

Animals were mounted on 7% agarose pads in M9 buffer containing 7.5 mM levamisole and covered with a coverslip. The synchronized mid or late L4 animals were used for imaging in panels B–C'', and young adult hermaphrodites were imaged in panels D-F.

**Generation of CRISPR knock-in strains**

CRISPR/Cas9 editing was performed using the Bristol N2 strain as the wild type. Synthetic crRNAs and tracrRNA were purchased from Horizon Discovery. Repair templates and single-stranded DNA oligos were synthesized by Integrated DNA Technologies (IDT). Approximately 20-30 young adult hermaphrodites were injected with the CRISPR/Cas9 injection mix. The N-terminal tag was amplified from the plasmid pDD282, which contains *C. elegans* codon-optimized eGFP. We co-injected the *dpy-10 ( cn64 )* roller marker with the GFP::PPIT-2 injection reagent (Arribere et al., 2014). The sequences for the *dpy-10 ( cn64 )* crRNA and single-stranded repair template (ssODN) have been added to the Reagents section/table. F1 roller progeny of injected animals were initially selected based on visible GFP expression and subsequently screened by single-worm PCR across the insertion boundaries using the primers: Forward 5' ccccctttgaaaactcacattt 3' and Reverse 5' gggttttaggccatctgtgga 3'. The amplification sequence is listed below:

ccccctttgaaaactcacatttataaattttgatttttggaaatgaattaattaatttctaatttgaactttcagctttattttgaaattttttttttaataatcgttaaaggtcaaattacgtataacagttggaattggtcaaatttttcaaaacaacttcattacttcgaaaaaaac **ATG** agtaaaggagaagaattgttcactggagttgtcccaatcctcgtcgagctcgacggagacgtcaacggacacaagttctccgtctccggagagggagagggagacgccacctacggaaagctcaccctcaagttcatctgcaccaccggaaagctcccagtcccatggccaaccctcgtcaccaccttctgctacggagtccaatgcttctcccgttacccagaccacatgaagcgtcacgacttcttcaagtccgccatgccagagggatacgtccaagagcgtaccatcttcttcaaggtaagtttaaacatatatatactaactactgattatttaaattttcaggacgacggaaactacaagacccgtgccgaggtcaagttcgagggagacaccctcgtcaaccgtatcgagctcaaggtaagtttaaacagttcggtactaactaaccatacatatttaaattttcagggaatcgacttcaaggaggacggaaacatcctcggacacaagctcgagtacaactacaactcccacaacgtctacatcatggccgacaagcaaaagaacggaatcaaggtcaacttcaaggtaagtttaaacatgattttactaactaactaatctgatttaaattttcagatccgtcacaacatcgaggacggatccgtccaactcgccgaccactaccaacaaaacaccccaatcggagacggaccagtcctcctcccagacaaccactacctctccacccaatccgccctctccaaggacccaaacgagaagcgtgaccacatggtcctcctcgagttcgtcaccgccgccggaatcacccacggaatggacgagctctacaagggagcatcgggagcctcaggagcatcgATTGTAAAGgtgagttattttttgatattggaagatggccgaagaattctatggtggcctaggaatccacagatggcctaaaaccc

Candidate insertions were validated by Sanger sequencing of both the 5′ and 3′ genomic junctions. All GFP::PPIT-2 lines used for imaging were established as homozygous knock-in stocks before imaging.

GFP::PPIT-2 CRISPR information:

Guide RNA: 5' CGAAAAAAACATGATTGTAA”

Repair template primer F1: 5' tttcaaaacaacttcattacttcgaaaaaaacATG agtaaaggagaagaattgttc 3'

Repair template primer R1: 5' tcttccaatatcaaaaaataactcacCTTTACAAT cgatgctcctgaggctcccgatgctcc CTTGTAGAGCTCGTCCATTC 3'

Genotype Primer F1: 5' ccccctttgaaaactcacattt 3'

Genotype Primer R1: 5' gggttttaggccatctgtgga 3' 

*dpy-10 ( cn64 )* CRISPR information:

Guide RNA: 5' GCTACCATAGGCACCACGAG”

Repair template: 5' CACTTGAACTTCAATACGGCAAGATGAGAATGACTGGAAACCGTACCGCATGCGGTGCCTATGGTAGCGGAGCTTCACATGGCTTCAGACCAACAGCCTAT 3'

***C. elegans* strains used in this study:**

**Table:** 

**Strain Name**	**Genotype**	**Source**
N2	Bristol wild-type	CGC
XFB7	*fasn-1 (xmb13[ fasn-1 ::rfp]) I *	This study
XFB143	*PPIT-2(xmb11[GFP::PPIT-2]) I*	This study

**Acknowledgments:**

We thank the Caenorhabditis Genetics Center (CGC), which is funded by the National Institutes of Health Office of Research Infrastructure Programs (P40OD010440), for providing strains for this study. We also thank members of the UF Worm community for insightful feedback and discussion.

**Funding:**

This work was supported by the National Institute of General Medical Sciences/National Institutes of Health under Awards Numbers R00GM145224 and R35GM162564-01 to X.F.B. and G.H., as well as startup funds from the University of Florida to the Bai laboratory.
